# Biogenic Silver Nanoparticles from Two Varieties of *Agaricus bisporus* and Their Antibacterial Activity

**DOI:** 10.3390/molecules27217656

**Published:** 2022-11-07

**Authors:** Abeer M. Al-Dbass, Sooad Al Daihan, Aisha A. Al-Nasser, Leenah Saleh Al-Suhaibani, Jamilah Almusallam, Bushra Ibrahem Alnwisser, Sarah Saloum, Razan Sajdi Alotaibi, Laila Abdullah Alessa, Ramesa Shafi Bhat

**Affiliations:** Biochemistry Department, College of Science, King Saud University, Riyadh 11495, Saudi Arabia

**Keywords:** *Agaricus bisporus*: antibacterial activity, silver nanoparticles

## Abstract

*Agaricus bisporus*, the most widely cultivated mushroom, is safe to eat and enriched with protein and secondary metabolites. We prepared silver nanoparticles (AgNPs) from two varieties of *A. bisporus* and tested their antibacterial activity The synthesized AgNPs were initially confirmed by UV-Vis spectroscopy peaks at 420 and 430 nm for white and brown mushrooms AgNPs, respectively. AgNPs were further characterized by zeta sizer, transmission electronic microscopy (TEM), Fourier transform infrared (FTIR), and energy-dispersive X-ray spectroscopy (EDX) prior to antibacterial activity by the well diffusion method against six bacterial strains which include *Staphylococcus aureus*, *Staphylococcus epidermis*, *Bacillus subtilis*, *Escherichia coli*, *Salmonella typhi*, and *Pseudomonas aeruginosa.* TEM results revealed a spherical shape with an average diameter of about 11 nm in the white mushroom extract and 5 nm in the brown mushroom extract. The presence of elemental silver in the prepared AgNPs was confirmed by EDS. The IR spectrum of the extract confirmed the presence of phenols, flavonoids, carboxylic, or amide groups which aided in the reduction and capping of synthesized AgNPs. The AgNPs from both extracts showed almost the same results; however, nanoparticles prepared from brown mushrooms were smaller in size with strong antibacterial activity.

## 1. Introduction 

Silver nanoparticles (AgNPs) are the most widely explored nanostructures in various fields of biomedicine [1]. In the past few years, nanostructures of silver are being used as antibacterial agents in the health sector, cosmetic industry, and food storage companies due to their unique morphology and small size in the range from 1 to 100 nm [2,3]. Metallic silver ions are activated through ionizations by the reduction process. Ionic silver can bind to the cell walls of microbes and can change the cell morphology. Nowadays AgNPs are considered next-generation antibiotics due to their broad-spectrum activity against many pathogenic microbes. The synergistic effect of AgNPs with commercial antibiotics is tested against resistant microbes to replace old-generation drugs [4]. The full mechanism behind the bactericidal effect of AgNPs is not explained yet but it may depend on the cation releasing capacity of nanoparticles (NPs) which can chelate DNA, disturb protein structure, and disrupt bacterial cell walls [5,6,7,8]. Silver ions possess electrostatic attraction against proteins and can enhance the permeability of the cytoplasmic membrane leading to the disruption of the bacterial envelope [9]. Inside the cell, these ions can deactivate respiratory enzymes to generate reactive oxygen species and disturb adenosine triphosphate production [9]. The production of reactive oxygen species is mainly responsible for cell membrane disruption and deoxyribonucleic acid (DNA) modification [10]. Additionally, silver ions can interrupt DNA replication and cell reproduction by reacting with the sulfur and phosphorus of DNA and stop protein synthesis by denaturing ribosomes in the cytoplasm [11]. The AgNPs interact with the cell wall of microbes in a shape-dependent manner. Triangular shape AgNPs have a better effective area of interaction with the membrane, compared to rod-shaped and spherical-shaped particles [12,13]. 

Different chemical and physical methods employed to fabricate nanoparticles have limitations [14]. The physical method needs costly equipment with high energy, pressure, and temperature [15,16,17,18]. The main problem faced by chemical methods is the use of harmful solvents and reducing agents with costly metal salts [19]. To overcome these challenges, researchers get encouraged by the biological approach to fabricating nanoparticles by using extracts from organisms such as plants [20], bacteria [21], fungi [22], and algae [23] for reducing metal salts to metal nanoparticles. The stability of these nanoparticles is enhanced by capping with biomolecules present in the organism [24]. This method is easy to handle and a bit economical compared to physical and chemical methods. 

Many studies have reported biological approaches to nanoparticle production from edible mushrooms [15,16,17,18,19,20,21,22,23,24,25,26,27,28]. Edible mushrooms are rich in protein and are enriched with essential amino acids. They contain a significant amount of secondary metabolites, such as terpenes, steroids, anthraquinones, benzoic acid derivatives, quinolones, and many vitamins such as B complex, C, D, and E [29,30,31]. Mushrooms belong to the fungi kingdom and have good nutritional and medicinal value as anti-cancer, antidepressant, antidiabetic and antimicrobial properties [31,32]. Many edible mushrooms are used as reducing and stabilizing agents in the green process of nanoparticle synthesis. The mushroom-based nanoparticle synthesis process has good yields and low toxicity due to protein-rich extracts [33,34]. The mushroom-based nanoparticles have a special coat that improves their life span and stability. During the synthesis process, the extracellular enzymes present in the mushroom extract also act as a stabilizing agent and help in reducing toxicity. In comparison to other biological sources, mushrooms have a higher metal binding capacity [35,36]. *Agaricus bisporus* is safe to eat and the most widely cultivated mushroom all over the world with two varieties, the white button mushroom and Portobello or the brown button mushroom [37]. In the present study, we use two varieties of *A. bisporus* for fabricating AgNPs. Aqueous extracts of white and brown mushrooms were used for the green synthesis of nanoparticles from silver salt and were further investigated for antibacterial properties. 

## 2. Results and Discussion 

### 2.1. Biosynthesis of the Prepared AgNPs

We investigated the biosynthesis of silver nanoparticles from two varieties of *A. bisporous* (white and brown mushrooms) and evaluated their antibacterial potential. The color change of the mushroom extract after adding silver nitrate solution was the first indication of the formation of silver nanoparticles due to the reduction process of silver ions. In the present study, the reducing agents were present in the mushroom extract as *A. bisporous* are rich sources of antioxidants mainly flavonoids [38]. Compared to other organisms, Fungi have some attributes for the biosynthesis of metallic nanoparticles due to the presence of large amounts of proteins and enzymes per unit of biomass [39]. 

### 2.2. Characterization of the Prepared AgNPs

**Figure 1:**
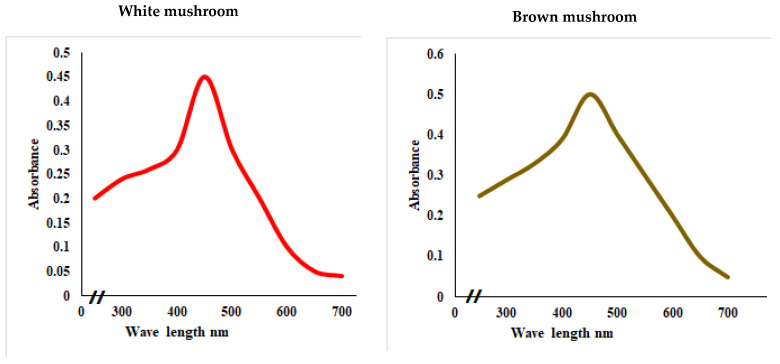
UV–visual spectra showing the absorbance peak of AgNPs prepared from white and brown mushroom.

**Figure 2:**
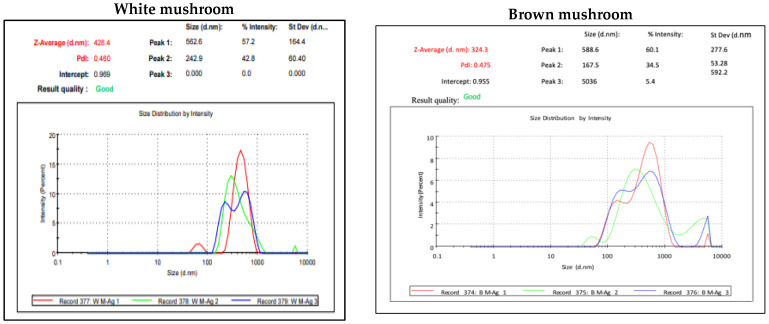
Dynamic light scattering (DLS) result for AgNPs prepared from white and brown mushroom.

**Figure 3:**
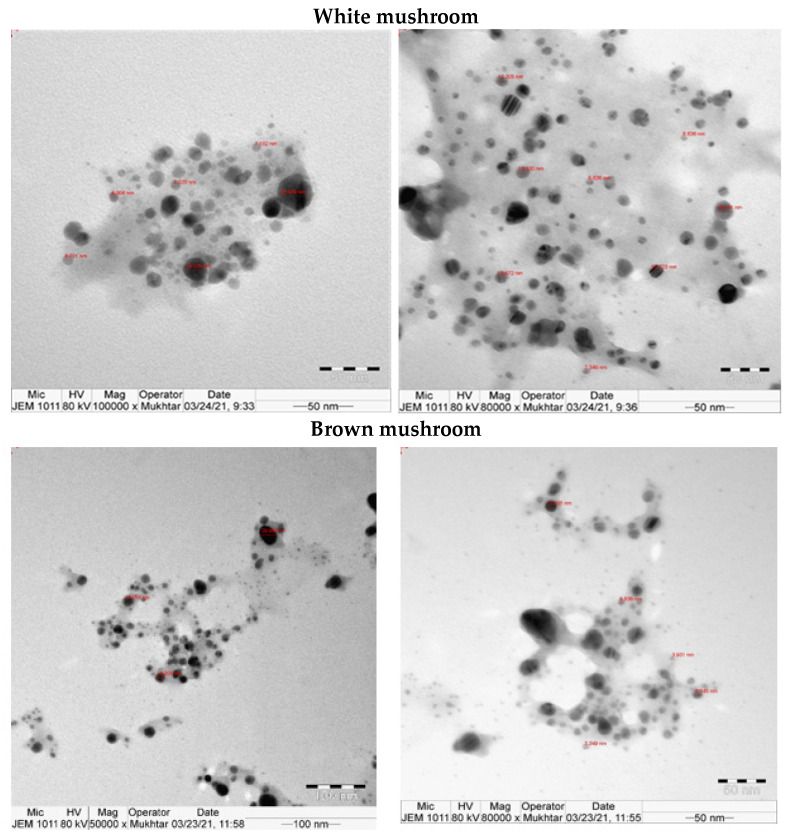
TEM for AgNPs prepared from white and brown mushroom.

**Figure 4:**
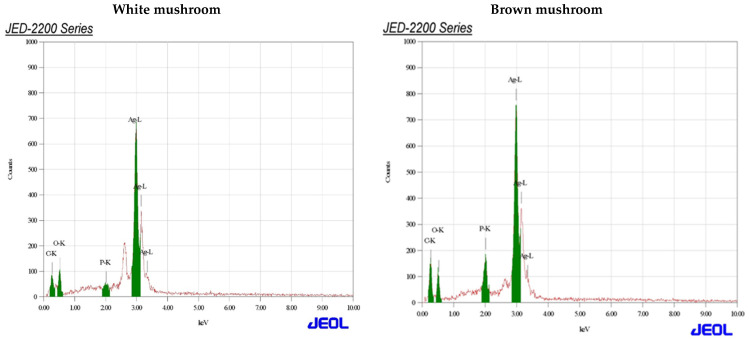
Energy-dispersive X-ray spectrum assay for AgNPs prepared from white and brown mushroom.

UV-vis absorbance spectra of silver nanoparticles of white and brown mushrooms were recorded at 420 and 430 nm, respectively, as shown in Figure 1, which confirms the presence of silver nanoparticles due to surface Plasmon resonance (SPR) electrons. Generally, silver nanoparticles have an absorption peak in the range of 400–450 nm depending upon their size [40] Our results point toward the formation of different particle sizes from two mushroom extracts. Silver nanoparticles possess free electrons and positively charged nuclei with a minute gap in between to allow the free movement of electrons. Mutual oscillation of these electrons generates an SPR absorption band in resonance with the light wave [41]. The pattern of SPR mainly depends on the size, and shape of metal nanoparticles, in addition to the dielectric properties of the medium used for the synthesis of nanoparticles. The Z-average mean size of white mushroom AgNPs was 428.8 d.nm, with a polydispersity index (PDI) of 0.480 while brown mushroom AgNPs recorded Z-average mean size of 324.3 d.nm with a PDI of 0.475, as shown in Figure 2. PDI value defines the average uniformity of nanoparticles in a solution. It also shows aggregation and consistency in nanoparticles all over the sample. PDI value less than 0.1 indicates monodispersion whereas the values above 0.7 specify a large size distribution. The shape and size of a nanoparticle reveal its physical and chemical properties. The size distribution is thus an important issue for procedures generating uniformly sized and shaped nanoparticles. Transmission electron microscopy (TEM) stands as one of the best techniques for exploring the size and shape of the nanoparticles and providing their size distribution [42]. The TEM image shown in Figure 3 confirms the formation of silver nanoparticles from the mushroom extracts. Particles were spherical in shape with an average diameter of about 11 nm in the white mushroom extract and 5 nm in the brown mushroom extract. Studies have reported that the UV–Vis spectra in a range of 390 nm–420 nm indicate spherical-shaped AgNPs which agreed with our results [41]. EDX was used to investigate the elemental composition of the biosynthesized AgNPs as shown in Figure 4. The formation of silver nanoparticles has been confirmed by EDX, having the highest peak at 3kev, confirming the presence of metallic silver nanocrystals. The Ag peak of Brown mushroom nanocomposite was found higher than that of White mushroom nanocomposite which may be due to the slight difference in the biomolecules present in both extracts which were confirmed by different peaks in the Infrared spectra. The presence of other elements including carbon, oxygen, and potassium was also observed in the spectrum which may serve as capping organic agents bound to the surface of the silver nanoparticles.

### 2.3. Fourier-Transformed Infrared (FTIR)

**Figure 5:**
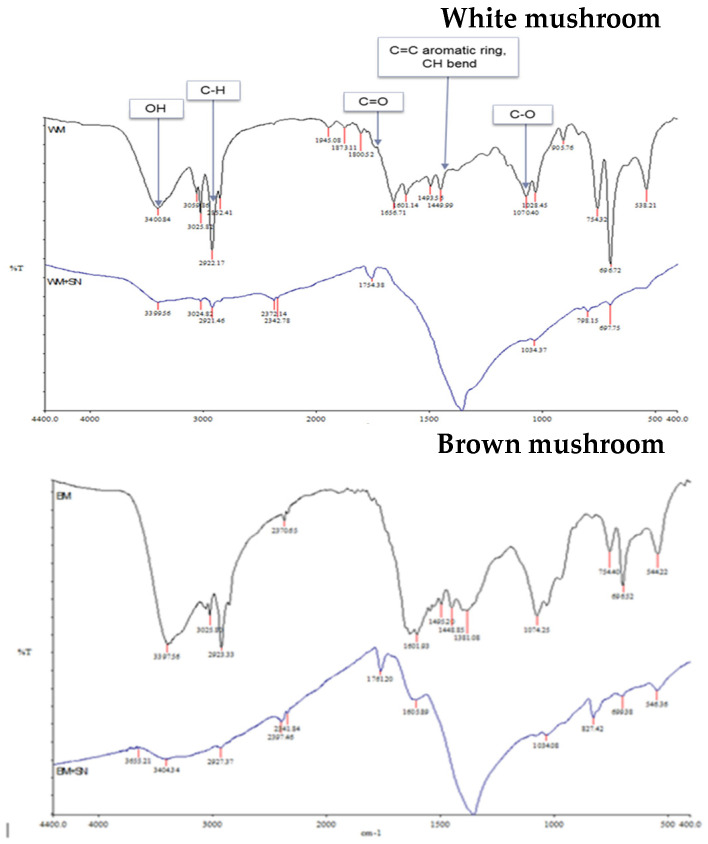
Infrared spectra of mushroom extracts and prepared AgNPs.

Mushrooms are rich in active compounds [30,31,32] which can assist the green synthesis of NPs and can act as a capping agent for less aggregation of synthesized nanoparticles [32]. FTIR spectroscopy was used to analyze these components in mushroom extracts (Figure 5). Although mushrooms are rich in bioactive compounds and nutrients, the contents of these bioactive compounds depend on the cultivar [43]. The recorded spectral of two different mushrooms shows some distinct spectral curves which indicate slightly different compositions in lipids (3000–2800 cm^−1^), proteins (1700–1500 cm^−1^), pectin (1790–1720 cm^−1^), cellulose and hemicellulose (1300–1180 cm^−1^) and other carbohydrates (fingerprint region 1200–900 cm^−1^) [44]. The biological method of synthesizing AgNPs is a three-step procedure. First is the activation phase where metal ions are reduced with the help of reducing agents present in the extract. The second is the growth phase where reduced metal ions form NPs. Finally capping is performed by metabolites present in the extract for stability [31]. The IR spectrum of the extract shows peaks corresponding to the OH stretching due to phenols, N-H and C-H bending corresponding to amide and aromatic groups, and peaks for C=C stretch. The IR spectrum of the synthesized nanoparticles shows a shift in the peaks and also the appearance of some new peaks. The presence of new peaks at 3399.56 cm^−1^; 2921.45 cm^−1^; 1754.38 cm^−1^; in white mushroom-AgNPs and at 3404.21 cm^−1^; 2141.54 cm^−1^; 1761.20 cm^−1^; 1605.39 cm^−1^; 1380.42 cm^−1^; 699.38 cm^−1^ in brown mushroom-AgNPs indicates some changes in the chemical composition and functional groups of proteins, lipids, and carbohydrates in the mushroom extract. The reduction and capping of synthesized AgNPs might be due to the presence of phenols, flavonoids, carboxylic, or amide groups which were seen in the IR spectrum.

### 2.4. Antibacterial Activity

Nowadays, many pathogenic bacteria are resistant to chemical disinfectants, hence AgNPs are being used as antibacterial agents in detergents and disinfectants [45]. Silver as such is considered a broad-spectrum antimicrobial agent but its nano form could be more useful due to its large surface area which increases microbial exposure area and time. 

**Figure 6:**
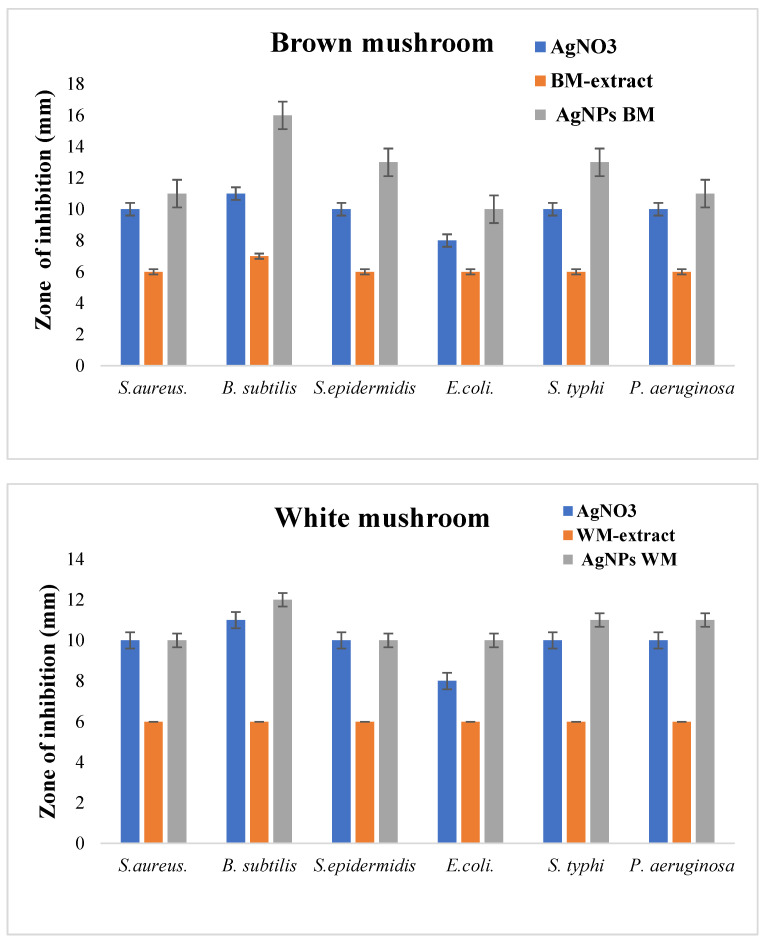
Antibacterial activity of white and brown mushroom extracts and prepared AgNPs against different bacterial strains.

In the present study, the antibacterial efficiency of AgNPs against six bacteria strains was evaluated. The AgNPs produced from brown mushrooms were more effective in inhibiting the growth of test bacteria as compared to AgNPs from white mushrooms (Figure 6). AgNPs of brown mushrooms were more potent against *B. subtilis* with the zone of inhibition of 16 mm, whereas for white mushrooms it was 12 mm as shown in Figure 6. Against *S. aureus, S. epidermis, E. coli, S. typhi*, and *P. aeruginosa* the zone of inhibition was 11 mm, 13 mm, 10 mm, 13 mm, and 11 mm, respectively, for AgNPs of brown mushroom and 10 mm, 10 mm, 10 mm, 11 mm, 11 mm, respectively, for AgNPs of white mushroom. These antibacterial efficacy NPs from brown mushrooms can be ascribed to their small size. The small NPs tend to be more toxic than large NPs due to the relatively larger surface area to volume ratio of smaller NPs as compared to larger ones. A larger surface area to volume ratio significantly upturn ROS levels thus damaging essential biomolecules such as DNA, proteins, and lipids [46]. Many previous studies have suggested the strong antimicrobial activity of nanoparticles of small size [47,48] Microorganisms have developed many systems to neutralize antibiotics and almost 65% of the antibiotics are ineffective against intracellular infections. The main reason is poor permeability and low intracellular retention due to their hydrophilic nature. In contrast, NPs can penetrate through the hydrophobic barrier, especially in phagocytic cells which engulf NPs and increase their intracellular activity [49]. We observed that synthesized AgNPs showed higher antibacterial activities against both gram-positive and gram-negative strains as compared to silver nitrate solution (Figure 6). Due to their small size, AgNPs can penetrate the thick and rigid cell walls of gram-positive bacteria and the strong lipopolysaccharide membrane of gram-negative bacteria [50,51]. AgNPs can interact with the genetic material (DNA) and other important constituents, thereby disturbing its integrity, which ultimately leads to cell death [52].

## 3. Materials and Methods

### 3.1. Mushroom Extract 

Two varieties of *A. bisporous* (white and brown mushrooms) were collected from Riyadh; 5 g of both samples were washed and blended in 500 mL of distal water separately and stirred for 24 h. The mixture was filtered and centrifuged at 6000 rpm for 10 min. The supernatant was collected and stored at 4 °C until further use.

### 3.2. Biosynthesis of AgNPs

The white and brown mushroom extracts were used as reducing agents for the synthesis of silver nanoparticles. Both extracts were added to a hot silver nitrate solution keeping the final concentration at 5 mM. The reduction of Ag ions to AgNPs was monitored through the color change from transparent to brown and light brown to dark brown for white and brown mushrooms, respectively, for 24 h. 

### 3.3. Characterization of the Prepared AgNPs

At first, prepared AgNPs were ascertained by UV-VIS spectrophotometer (1800 UV Shimadzu) by measuring absorbance in the range of 200–700 nm. UV-VIS spectroscopy is the quickest method for the detection of AgNP as The nanoAg can shift the absorption maximum to the visible light region; 1ml of the mixture was monitored till the completion of bioreduction of Ag ion aqueous solution by scanning in UV-visible (vis) spectra.

The hydrodynamic size of the AgNPs was measured by the dynamic light scattering (DLS) technique on a zeta Sizer NANO (Malvern). This technique illuminates particles with a Wavelength of 633 nm at a red laser with a scattering angle of 173 and a medium viscosity of 0.887 mPa at 25 °C to measure particle size. An electron beam to image an AgNPs sample on TEM-1011 was used to identify and measure the nanoparticle size and its morphology. Transmission Electron Microscopy (TEM) is a technique used for particle sizing. It helps sample imaging on the absorption of a beam of electrons, during its passage through an ultrathin sample (less than 100 nm). The transmitted beam is then projected onto a detector, which enables the visualization of objects in the nanometer-sized range. The elemental composition of nanoparticles was determined by the energy dispersive spectrum (EDX). It is a technique used for the chemical characterization of materials based on the emission of specimen characteristic X-rays. Spectrum peaks are correlated with the elemental composition of the sample. 

### 3.4. Fourier Transformed Infrared (FTIR)

All the samples were dried and encapsulated in Potassium Bromide for analysis of functional groups by using Perkin Elmer FTIR-Spectrometer Spectrum (Spectrum BX) in the range from 4000 to 400 cm^−1^.

### 3.5. Antibacterial Activity 

The antibacterial activity of prepared AgNPs and mushroom extracts were tested against six bacterial strains by well diffusion method. The bacterial strains used in this study were provided by the central laboratory at King Saud University which includes *Staphylococcus aureus, Staphylococcus epidermis, Bacillus subtilis, Escherichia coli, Salmonella typhi,* and *Pseudomonas aeruginosa*. The well diffusion method was used to test the antibacterial activity of the mushroom extracts; nano Au particles and silver nitrate solution were placed in 6 mm wells in the nutrient agar. The plates were swabbed with the microbial cultures and wells were filled with the samples and incubated at 37 °C for 16 h. At the end of the incubation period, the maximum zone of inhibition was observed and measured for analysis against each type of microorganism.

## 4. Conclusions

*Agaricus bisporus* extract acts as a reducing and stabilizing agent for fabricating silver nanoparticles. White and brown mushrooms showed almost the same results; however, nanoparticles prepared with brown mushrooms were smaller in size with strong antibacterial activity. 

## Data Availability

Data is contained within the article.
